# The influence of 3D renal reconstruction on surgical planning for complex renal tumors: An interactive case-based survey

**DOI:** 10.1590/S1677-5538.IBJU.2022.0623

**Published:** 2023-04-05

**Authors:** Raed A. Azhar

**Affiliations:** 1 Department of Urology Faculty of Medicine King Abdulaziz University Jeddah Saudi Arabia Department of Urology, Faculty of Medicine, King Abdulaziz University, Jeddah, Saudi Arabia

**Keywords:** Neoplasms, Nephrectomy, Surgical Procedures, Operative

## Abstract

**Objectives:**

To evaluate the role of three-dimensional (3D) reconstruction in preoperative planning for complex renal tumors.

**Materials and Methods:**

A well-planned questionnaire was distributed among the attending urologists at an international meeting. The questionnaire inquired about demographic data, surgical experience, partial nephrectomy (PN) versus radical nephrectomy (RN), surgical approach, time of ischemia, probability of postoperative urine leakage and positive surgical margins after viewing computed tomography (CT) scans and their respective 3D models of six complex renal tumors. Following the CT scans, attendees were asked to view randomly selected reconstructions of the cases.

**Results:**

One hundred expert urologists participated in the study; 61% were aged between 40 and 60 years. Most of them (74%) were consultants. The overall likelihood of PN after viewing the 3D reconstructions significantly increased (7.1±2.7 vs. 8.0±2.2, p<0.001), the probability of conversion to RN significantly decreased (4.3±2.8 vs. 3.2±2.5, p<0.001), and the likelihood of urine leakage and positive surgical margins significantly decreased (p<0.001). Preference for the open approach significantly decreased (21.2% vs. 12.1%, p<0.001), while selective clamping techniques significantly increased (p<0.001). After viewing the 3D models, low expected warm ischemia time and estimated blood loss were significantly preferred by the respondents (p<0.001). Surgical decision change was significantly associated with performance or participation in more than 20 PNs or RNs annually [3.25 (1.98-5.22) and 2.87 (1.43-3.87), respectively].

**Conclusions:**

3D reconstruction models play a significant role in modifying surgeons’ strategy and surgical planning for patients with renal tumors, especially for patients with stronger indications for a minimally invasive and/or nephron-sparing approach.

## INTRODUCTION

Minimally invasive partial nephrectomy is currently considered the best option for the management of localized small renal tumors (1). Patient and tumor characteristics, such as the anatomic location and extension of the tumor within the kidney and its relationship with other structures, may influence surgical decision-making and the choice of the appropriate surgical approach (2). It is difficult to characterize anatomical structures using only two-dimensional (2D) images, including computed tomography (CT) and magnetic resonance imaging (MRI).

Three-dimensional (3D) printing is a promising technology that creates specific 3D printed models based on routine CT or MR imaging data. This technique can accurately replicate complex anatomical structures and pathology and improve surgical planning and understanding of the complexity of different lesions (3, 4). Consequently, this image manipulation helps to enhance surgical decisions, increases surgeon confidence, and minimizes perioperative complications (4). Early adoption of this 3D printing technology has revolutionized clinical practice and allowed surgeons to explain their technical procedures to patients before obtaining informed consent (5). This is particularly important because most renal masses are incidentally discovered, and patients may have a limited understanding of the unexpected diagnosis and ability to interpret CT images and their need for surgery.

Sun and Liu reported that 3D-printed kidney models have high accuracy in delineating renal tumors and surrounding structures and can significantly help in the preoperative planning and simulation of surgical nephrectomy (6). Moreover, in their feasibility study, Kyung et al. confirmed that 3D-printed kidney models developed to improve patients’ satisfaction were secondary to a better understanding of their disease. In addition, 3D models can improve surgical outcomes because of their aid in the appropriate surgical planning and orientation of the target tissue and prediction of postoperative renal function (7).

Furthermore, due to superior visualization of anatomical details and pathologic morphology, customized interactive virtual 3D models may help junior surgeons with training and enhance the operative skills of senior surgeons (8). Therefore, the purpose of the present study is to identify the role of 3D reconstruction as part of the preoperative planning process for complex renal tumors.

## MATERIALS AND METHODS

A well-planned questionnaire was distributed among the attending urologists at an international meeting after ethical approval number 108-23 had been obtained. The questionnaire collected information that included demographic data, surgical experience, partial versus radical nephrectomy, surgical approach, time of ischemia, and probability of postoperative urine leakage and positive surgical margins after viewing the CT scans and their respective 3D models of six complex renal tumors. Selected patients underwent partial nephrectomy by a single fellowship-trained surgeon. The attendees were asked to view the CT scans first, and then the respective 3D reconstructions of the patients’ kidneys were randomly displayed.

The survey consisted of two main sections. The first section assessed the baseline characteristics of the surgeons, including geographical region, age, sex, current level of training, years of practice, surgical approach frequently used in real practice, number of nephrectomy procedures performed or participated in annually, and previous experience in using the 3D models for preoperative planning. The second section assessed the clinical cases separately according to the CT and 3D models. For each case, respondents were asked about the likelihood of partial nephrectomy (PN), the probability of converting to RN, preferred approach, clamping technique, expected warm ischemia time and blood loss, and likelihood of urine leakage and positive surgical margin. For each clinical scenario, the responses were compared between the CT and 3D models. Finally, the respondents were asked whether they planned to use 3D virtual models in their practice (Supplementary material, Appendix 1).

### Surveyed cases

All presented cases included single renal tumors with no major vascular thrombosis or lymphadenopathy. All cases were managed by robotic transperitoneal nephrectomy, with warm ischemia, and all showed negative surgical margins. There were no intraoperative or postoperative complications, and none of the cases needed a blood transfusion. Most cases had an intermediate-complexity RENAL nephrometric score.

### Production of the 3D models

The CT scans were uploaded in DICOM format to the innovation laboratory’s website. By utilizing the laboratory’s technology, the images were reconstructed into 3D virtual interactive models that can be viewed using a web browser across a wide range of platforms.

### Data analysis

Data were analyzed using the commercially available Statistical Package for the Social Sciences software (SPSS Inc., Chicago, IL, USA), version 23. Categorical variables are presented as frequencies and percentages and were compared with Fisher’s exact test. Continuous variables are presented as the means and standard deviations and were compared with Student’s t test. Changing surgical planning for the displayed cases was assessed by multivariate logistic regression analyses. Two-tailed p values of less than 0.05 were considered statistically significant.

## RESULTS

### Demographics and practice patterns

The survey was completed by one hundred urologists with different levels of training, and 61% of the urologists were aged between 40 and 60 years. Most of them (74%) were consultants, and 53% were practicing in the KSA. Fifty-one percent were academics, and 71% of them had been in urology practice for more than 10 years. Fifty-nine percent of respondents had formally trained in minimally invasive surgery using laparoscopic (60%) and robotic (52%) surgical approaches, whereas 66% were involved in the surgical theater 2-3 days a week. Seventy percent and 37% of survey participants performed/assisted in 20-79 PNs and RNs annually, respectively, while 54% had previously used the 3D models for preoperative planning (Table-1). The tumor characteristics of the included cases are summarized in Table-2.

**Table 1:** - Demographic characteristics and clinical practice of all participants.

Variable (n=100)		No = %
Location of practice	Asia	67
	North America	15
	South America	10
	Europe	8
Age/years	<40	33
	40-60	61
	>60	6
Level of training	Fellow	6
	Specialist	20
	Consultant/Faculty	74
Years practicing Urology	<10	23
	10-20	46
	>20	31
Current job title or role	Clinical Fellow	6
	Registrar/Senior Registrar	20
	Consultant	74
Subspecialty	Minimally invasive	59
	Transplantation	6
	Uro-oncology	6
	General Urology	53
	Not applicable	36
Practice setting	Academic	51
	General hospital	46
	Private (Self-employed)	12
	Military hospital	31
	Tertiary care Center	12
Surgical approach frequently used/participated in practice	Open	5
	Laparoscopic	42
	Robotic	53
Days/week involved in the surgical theatre	One day	34
	2-3 days	66
Number of partial/radical nephrectomies performed or participated in annually	<20	30/58
	21-40	53/24
	51-80	17/13
	>80	0/5
Have you ever used 3D models for preoperative planning before	Yes	54
	No	41
If yes, then how many?	<10	36
	11–20	64

**Table 2:** Overall demographic and tumor characteristics of the surveyed cases.

Case	Age (y)	BMI kg/m^2^	Side	RENAL	Tumor size (cm)	EBL (mL)	Stage	Exophytic	Extension			WI
									Sinus	CS	Outside kidney	Time (min.)
Case 1	56	32	Right	6p	1.5	200	T1aNx	Yes	No	No	No	14
Case 2	58	30	Left	8ah	2.2	50	T1aNx	Yes	Yes	No	No	14
Case 3	48	31	Left	7p	2.2	75	T1aNx	Yes	No	No	No	14
Case 4	56	31	Right	8a	3.2	200	T3aNx	Yes	No	No	A major vein	16
Case 5	39	33	Left	11a	6.0	150	T1bNx	No	No	No	No	23
Case 6	25	24	Left	9p	2.6	100	T1aNx	Yes	No	No	No	17

CS = collecting system; EBL = estimated blood loss; WI = warm ischemia

### Clinical case decisions

**Figure 1:**
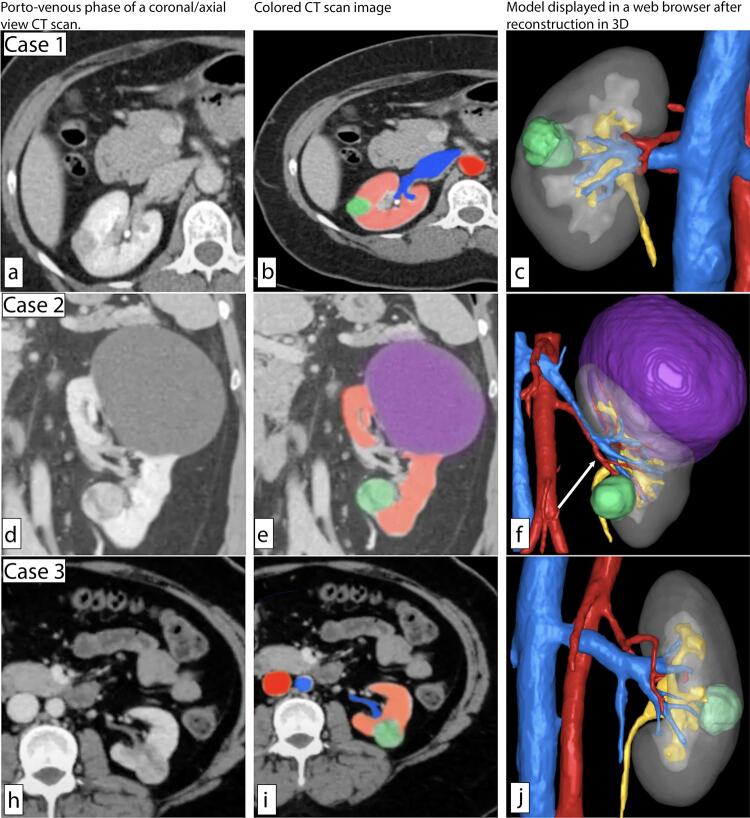
Representative CT scans and 3D reconstructions of the first three surveyed cases. Left, portovenous phase of a coronal/axial view CT scan. Middle, Colored CT scan image, red (artery/renal cortex), blue (vein), green (mass). Right, Model displayed in a web browser after reconstruction in 3D, red (artery), blue (vein), green (mass), purple (cyst), yellow (collecting system/ureter).

**Figure 2:**
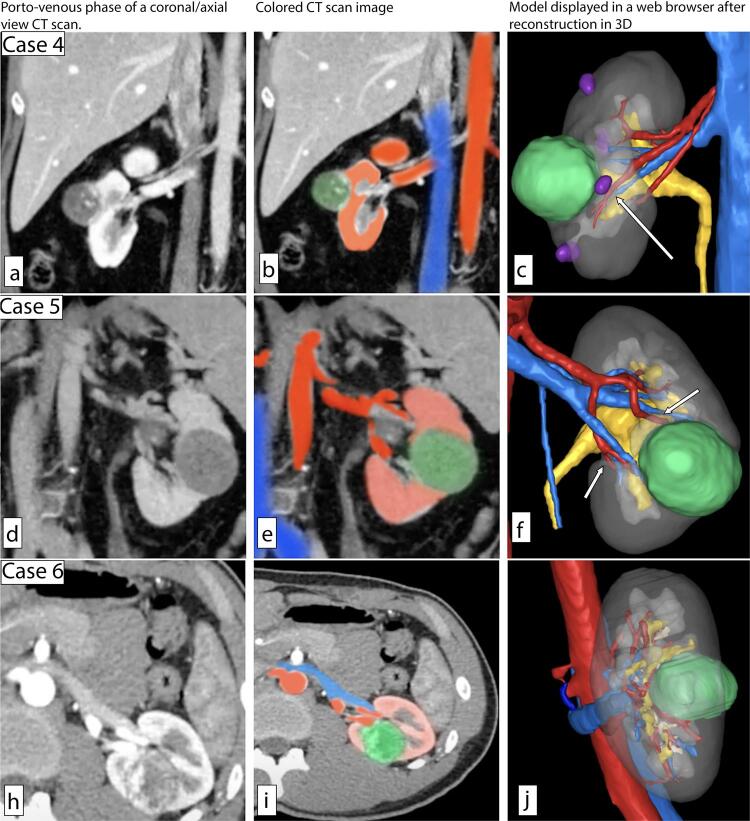
Representative CT scans and 3D reconstructions of the first three surveyed cases. Left, portovenous phase of a coronal/axial view CT scan. Middle, Colored CT scan image, red (artery/renal cortex), blue (vein), green (mass). Right, Model displayed in a web browser after reconstruction in 3D, red (artery), blue (vein), green (mass), purple (cyst), yellow (collecting system/ureter).

Table-3 shows the overall and case-by-case comparison of responses after the urologists had viewed the CT images and their respective 3D model reconstructions (Figures 1 and 2). After the urologists viewed the 3D reconstructions, the likelihood of selecting PN increased for all cases, and this was statistically significant in 4/6 of the cases. Additionally, the probability of conversion to RN significantly decreased in 5/6 of the cases. Responses indicating preference for the open surgical approach decreased with increasing preference for the minimally invasive approach in all cases; however, the responses were significantly different in 3/6 of the cases. Out of six cases, five cases were significantly associated with preferred selective clamping techniques, while 3/6 of the cases were significantly associated with decreased hot ischemia time and lower estimated blood loss (EBL). The probability of urine leakage and positive surgical margins were significantly decreased in 5/6 of the cases (Table-3).

**Table 3:** Overall and case-by-case comparison of responses after viewing of the CT images and their respective 3D model reconstructions.

Questions 23-30		Case 1 CT/3D	Case 2 CT/3D	Case 3 CT/3D	Case 4 CT/3D	Case 5 CT/3D	Case 6 CT/3D	Overall CT/3D
Likelihood of PN	Mean± SD	5.8±2.5/7.9±2.6	9.1±.5/9.2±1.0	8.4± 1.7/8.8±1.8	5.8±3.0/7.5± 2.3	5.7± 2.5/6.9± 2.4	7.1± 2.1/8.4± 1.8	7.1± 2.7/8.0± 2.2
	p value	<0.001	0.62	0.14	<0.001	0.002	<0.001	<0.001
Probability of conversion to RN	Mean± SD	5.9± 2.5/4.2± 2.8	2.4±2.0/1.9±1.4	2.7± 1.9/2.4± 2.0	5.5± 2.8/3.9± 2.6	5.4± 2.7/4.1± 2.4	3.9± 2.2/2.9± 2.2	4.3± 2.8/3.2± 2.5
	p value	<0.001	0.03	0.21	<0.001	0.001	0.003	<0.001
Preferred approach	Open	32/17	15/9	13/6	22/15	25/16	17/9	124/72
	Robotic	51/70	60/72	67/72	55/69	49/68	59/75	341/426
	Laparoscopic	15/12	25/19	17/21	21/16	26/16	24/16	120/96
	p value	0.02	0.18	0.21	0.17	0.02	0.04	<0.001
Preferred clamping technique	No clamping	2/9	16/16	8/11	5/5	1/2	3/7	35/50
	Artery alone	54/48	64/61	61/62	44/44	54/44	53/60	330/319
	Artery+ vein	34/34	17/16	26/16	43/30	42/40	39/21	201/157
	Selective	10/9	3/7	5/11	8/21	3/14	5/12	34/74
	p value	0.39	0.64	0.16	0.04	0.3	0.02	<0.001
Expected warm ischemia time (min)	< 10	3/14	40/40	15/30	8/11	4/5	8/19	78/119
	11–20	47/51	48/51	70/59	48/59	39/49	56/60	308/329
	> 20	50/35	12/9	15/11	44/30	57/46	36/21	214/152
	p value	0.003	0.77	0.04	0.12	0.29	0.01	<0.001
Expected blood loss (mL)	<200	33/49	77/81	65/81	40/44	32/36	43/67	290/358
	200–400	42/44	19/18	20/17	47/43	50/55	48/32	226/209
	> 400	25/7	4/1	5/2	13/3	18/9	9/1	74/23
	p value	<0.001	0.38	0.25	0.03	0.18	<0.001	<0.001
Likelihood of urine leakage	Mean± SD	4.8±2.3/3.7± 2.3	2.1± 1.5/2.0± 1.4	3.3± 2.0/2.4/1.7	4.8± 2.5/3.5± 2.1	5.3± 2.4/4.5± 2.2	4.1± 2.0/3.0± 2.1	4.1± 2.4/3.2± 2.1
	p value	0.001	0.42	0.001	0.02	0.02	<0.001	<0.001
Likelihood of positive surgical margin	Mean± SD	4.0± 2.2/3.1± 2.1	2.0± 1.4/1.8± 1.2	2.7± 1.7/2.0± 1.2	4.1± 2.5/2.8± 1.8	4.3± 2.1/3.4± 2.0	3.1± 1.6/2.4± 1.6	3.3± 2.1//
	/	/	/	/	/	/	/	/

CT = CT scan images; 3D = reconstructed 3D models; RN = radical nephrectomy

(a, b) Right 1.5 cm posterior mid-pole renal mass, (d, e) left 2.2 cm anterior lower-pole hilar renal mass with large upper pole simple renal cyst, (h, i) left 2.2 cm posterior mid-pole renal mass, (c, f, j) reconstructed 3D models with arrows indicating potential selective clamping arteries.

(a, b) Right 3.2 cm posterior mid-pole renal mass, (d, e) left 6 cm anterior mid-pole hilar renal mass, (h, i) left 2.6 cm posterior mid-pole renal mass, (c, f, j) reconstructed 3D models with arrow pointing toward a selective clamping artery.

The overall likelihood of selecting PN after viewing the respective 3D reconstructions significantly increased (7.1±2.7 vs. 8.0±2.2, p<0.001), while the probability of conversion to RN significantly decreased (4.3±2.8 vs. 3.2±2.5, p<0.001), and the likelihood of urine leakage and positive surgical margin significantly decreased (p<0.001) (Table-3). Preference for the open surgical approach decreased (21.2% vs. 12.1%, p<0.001), and an increased preference for the robotic approach was observed. The preferred clamping techniques significantly changed in favor of no clamping and selective clamping techniques (p<0.001). The expected warm ischemia time significantly changed after observing the 3D models, with an increasing low ischemia time of <10 min (13% vs. 19.8%) and a decreasing ischemia time of >20 min (35.7% vs. 25.3%) reported. Similarly, the estimated EBL significantly changed after the 3D models were observed; the percentages of EBL<200 mL and >400 mL were 49.1% vs. 60.7% and 12.5% vs. 3.9% (p<0.001), respectively (Table-3).

After correcting for baseline characteristics, changing the surgical indication for the displayed cases was not significantly associated with surgeon-related factors, including > 10 years in practice [OR (95% CI): 1.87 (0.92-2.21)], consultant job title [1.56 (0.89-1.94)], academic practice setting [1.23 (0.85-1.54)] or ≥2 days weekly in the surgical theatre [0.98 (0.66-1.08)]. Only a surgical decision change was significantly associated with performance or participation in more than 20 PNs or RNs annually [3.25 (1.98-5.22) and 2.87 (1.43-3.87), respectively].

## DISCUSSION

Most cases of PN with preserved kidney function have been shown to be effective and safe (9). With the advancement of nephron-sparing surgery toward larger lesions, the procedure itself has become much more complex (10). A trifecta achievement is seen as the optimal result when someone has undergone a partial nephrectomy. Bai and colleagues concluded that larger tumor sizes and medium and high PADUA scores are linked to lower odds of success in experiencing a trifecta (11). Cancer staging systems do not account for all possible variables in determining an individual’s prognosis and therefore cannot provide a complete picture of the patient’s needs. Moreover, some patients may have different outcomes even if they are at similar stages of the disease. Furthermore, they do not consider other factors, such as biomarkers and behavioral factors, that may be helpful in determining the prognosis (12).

Preoperative imaging plays a crucial role in surgical decision-making and patient counseling for major urological procedures, and novel 3D imaging models may challenge the data obtained from traditional 2D imaging studies. Patient-specific 3D models may overcome the limitations of traditional 2D imaging studies in addition to being valuable for patient counseling and conferring understanding of the pathology and planned surgical procedure (13). Three-dimensional printing technology has been applied in kidney surgery, including PN and flexible ureterorenoscopy, where knowledge of the intrarenal anatomy is critical for minimally invasive approaches. In addition, optimizing the surgical steps of the procedure by using these tools can improve perioperative and functional outcomes in cases with complex renal tumors (14, 15).

Grosso and colleagues demonstrated that 3D virtual models can promisingly assess surgical planning; the more complex the mass, the more advantages this reconstruction offers. These tools may boost tumor PN selection for complex renal masses (16). The current study aimed to evaluate the role of 3D virtual reconstruction in preoperative planning for complex renal tumors. The participating urologists significantly changed their surgical plans for all cases after viewing the 3D models that were reconstructed from relevant CT scans. In terms of the individual cases, the questioned parameters were significantly changed between 50% (3/6) and 83% (5/6) of the cases; these changes were made in favor of a minimally invasive approach, selective clamping technique, lower probability of conversion to RN, lower hot ischemia time and EBL and decreased probability of postoperative urine leakage and positive surgical margins. Overall, the significant changes approved for all these parameters also supported the individual case findings. This is consistent with the real scenarios performed in the investigated clinical cases in the current study, in which all cases underwent robotic PN by establishing a safety margin while minimizing hot ischemic time. Michiels et al. confirmed the impact of 3D kidney models in increasing the use of no clamping or selective segmental renal artery clamping and minimizing ischemia time, resulting in preservation of postoperative renal function (17).

Previous studies have shown that ischemia time and the proportion of preserved renal parenchyma influence postoperative renal function and filtration rate (18, 19). In three patients with complex renal tumors and unusual anatomy for which nephron-sparing surgery was indicated, Amparore et al. found that 3D virtual model guidance allowed surgeons to plan robotic PN based on preoperative visualization of the anatomical characteristics of the kidney and tumor (20).

The 3D technology necessary to facilitate robotic PN has become more available and less expensive, especially with the increased availability of advanced computer programs and printing material. Scott et al. described a process to create reproducible 3D kidney models that cost an average of 30 USD, and they suggested that these models are so cost-effective that they will become the standard of care for PN (21). Shirk et al. randomly assigned 48 patients undergoing robotic-assisted PN to control or intervention groups, according to surgical planning with CT and/or MR imaging with or without supplementary 3D models. Patients whose surgical planning involved 3D models had reduced operative and ischemia times, EBL, and length of hospital stay (22). However, these results should be cautiously interpreted in terms of an appropriate explanation of the odds ratios.

It is evident that perfect awareness of the intrarenal vascular anatomy would minimize the hot ischemia time during minimally invasive partial nephrectomy, thereby enhancing the complete and successful removal of the tumor while preserving the functioning of the renal parenchyma (23, 24). Although surgeons are usually concerned about these parameters, they should be cautious to avoid possible mismatches between the actual anatomy and 3D model (23).

In the present study, changing the surgical plan was only significantly associated with performance or participation in more than 20 PNs or RNs annually. This is consistent with the findings of Bertolo et al. (25), where respondents’ opinions changed regardless of their surgical experience. However, the latter study included expert urologists, urologists, and residents in urology and only compared their levels of expertise; the study did not consider the number of relevant procedures performed. In that study, regardless of surgeon experience, the authors found decision changes in more than 25% of cases after reviewing the 3D reconstruction, regardless of the experience level. It seems that performing or assisting in a given surgical procedure precisely improves the surgeon’s decision-making and planning abilities for such interventions.

The current survey may be limited by selection and recall biases. Such limitations are expected in any survey design and may limit the generalizability of the results. Participants may have been more inclined to participate due to their interest, and they may have overestimated the number of procedures performed. A higher number of decisions needed by the respondents for the six clinical case scenarios may compensate for the limited number of participants. Nevertheless, the findings of this study support the clinical and experimental data, which increasingly encourage the use of 3D reconstruction models for surgical planning in patients undergoing minimally invasive kidney surgery.

## CONCLUSION

Customized interactive virtual 3D models seem to provide superior visualization of the anatomical details and pathologic morphology of complex renal tumors over traditional visualization methods. Therefore, the surgeon can appropriately plan and modify the proposed surgical strategy, especially when minimally invasive partial nephrectomy is considered.
